# Supplementary material for: A new vulnerability to BET inhibition due to enhanced autophagy in BRCA2 deficient pancreatic cancer
Source: Cell Death Dis. 2023 Sep 21;14(9):620. doi: 10.1038/s41419-023-06145-9 (PMC10514057; doi:10.1038/s41419-023-06145-9)

Supplementary Figure 1

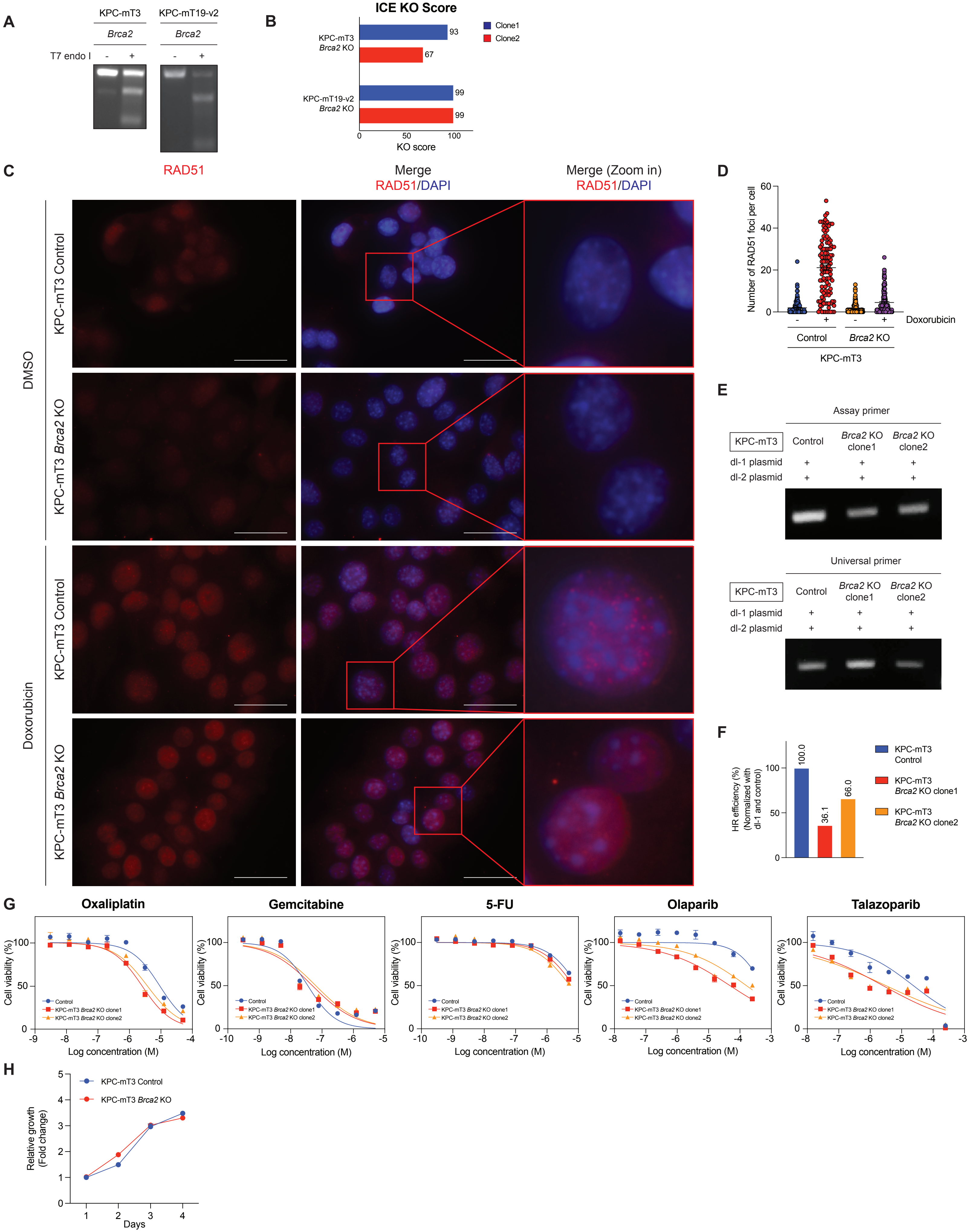

Supplementary Figure 2

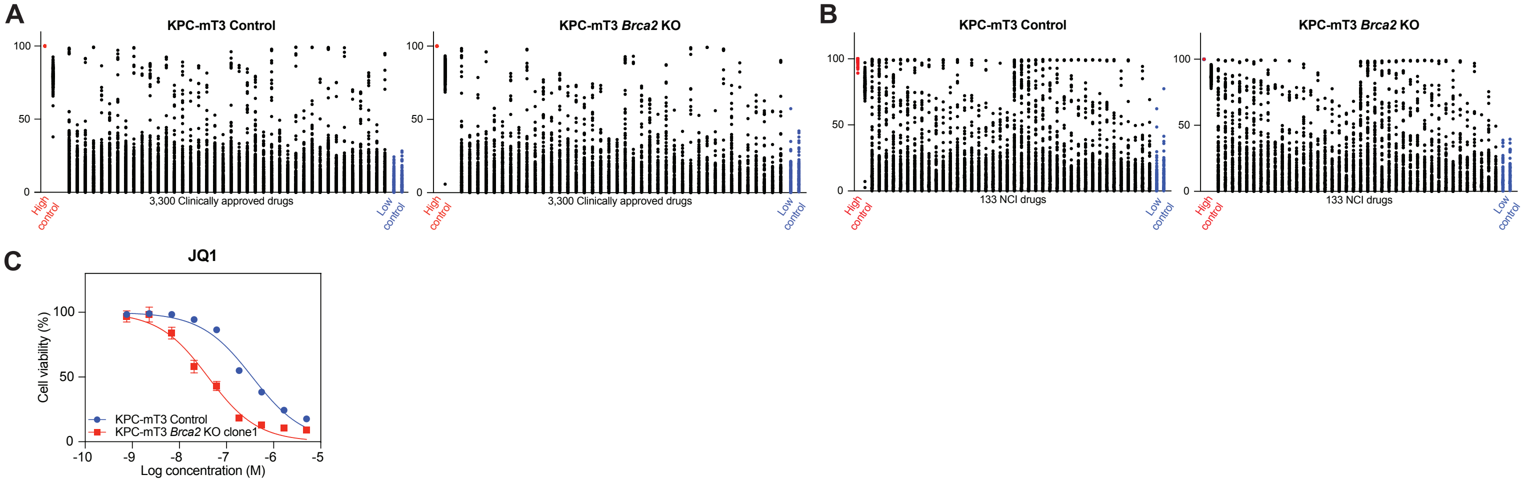

	Control	Brca2 KO
**HillSlope**	-1.223	-0.9907
**IC50**	1.880e-007	2.862e-008

Supplementary Figure 3

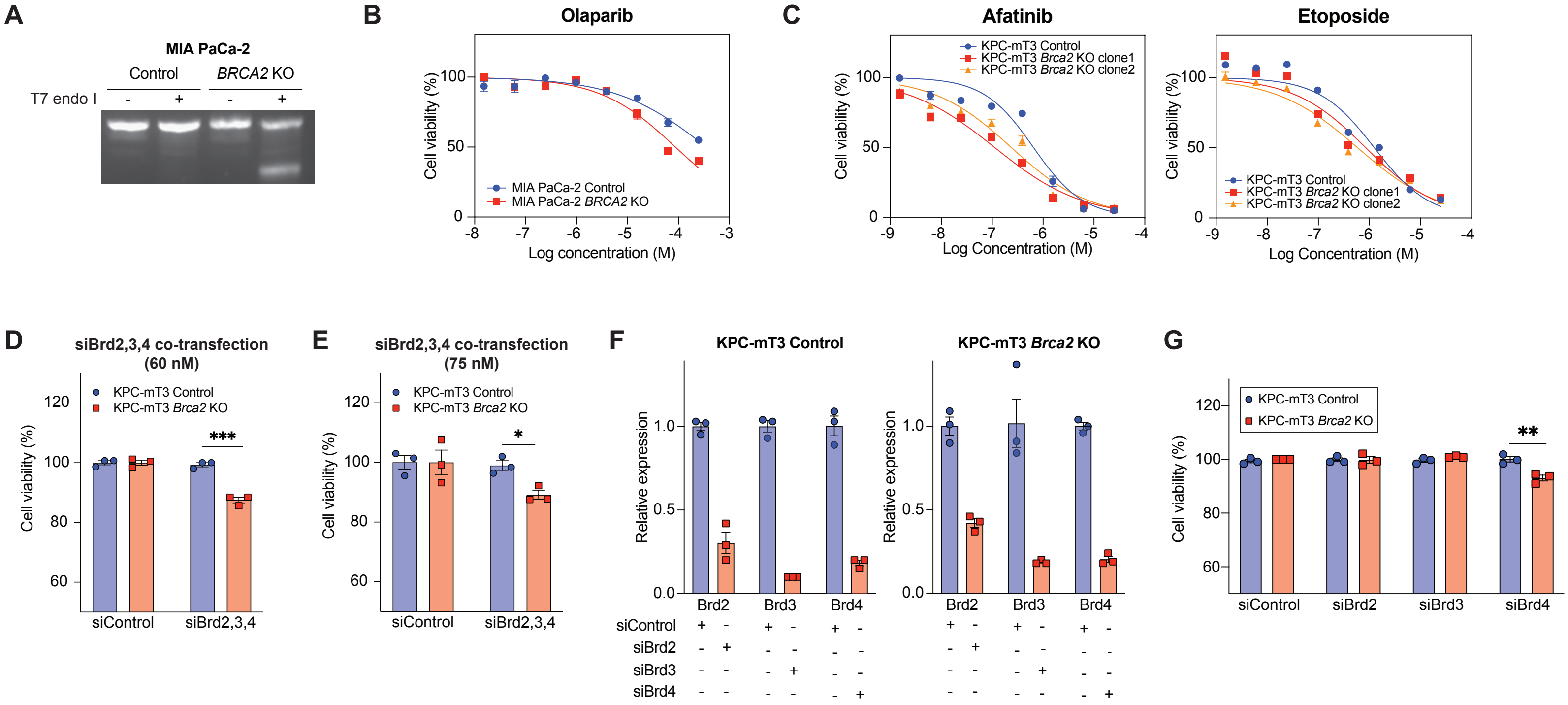

Supplementary Figure 4

A

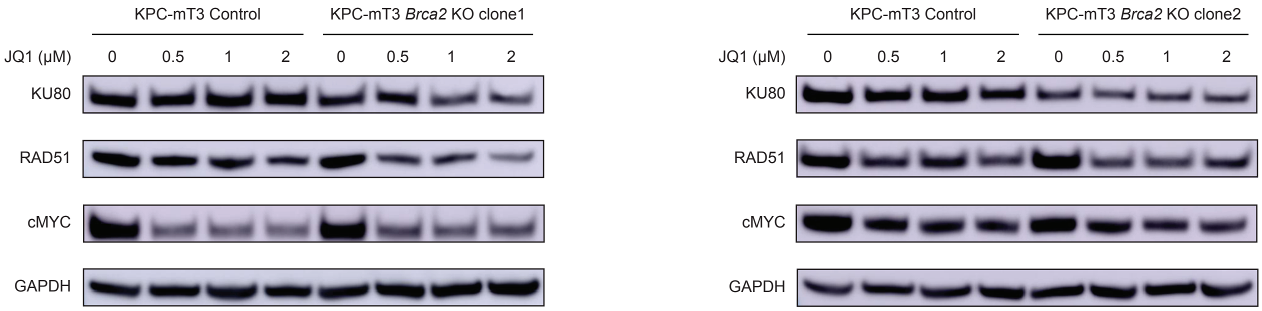

B

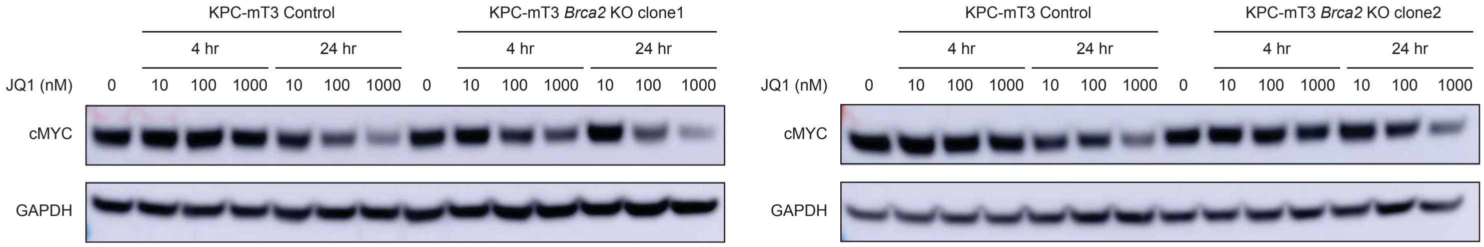

Supplementary Figure 5

A

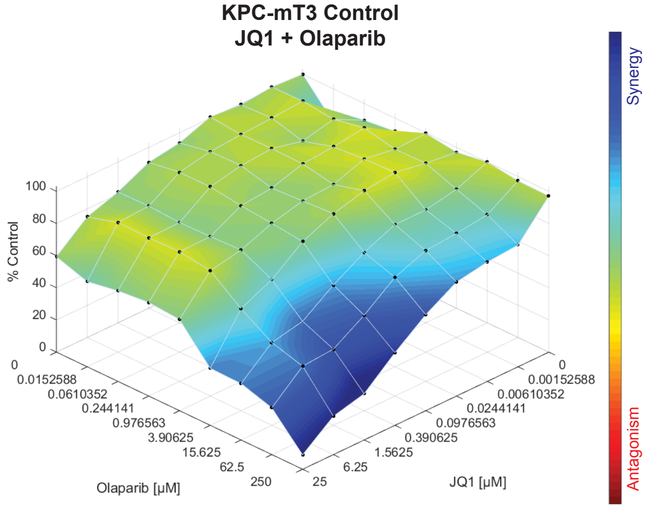

B

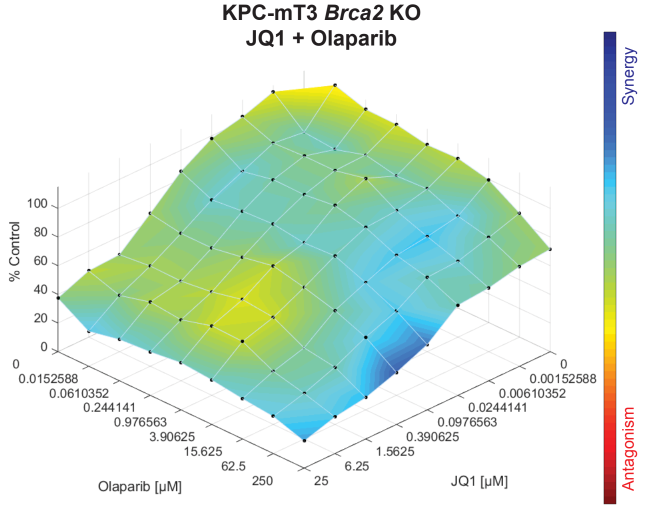

Supplementary Figure 6

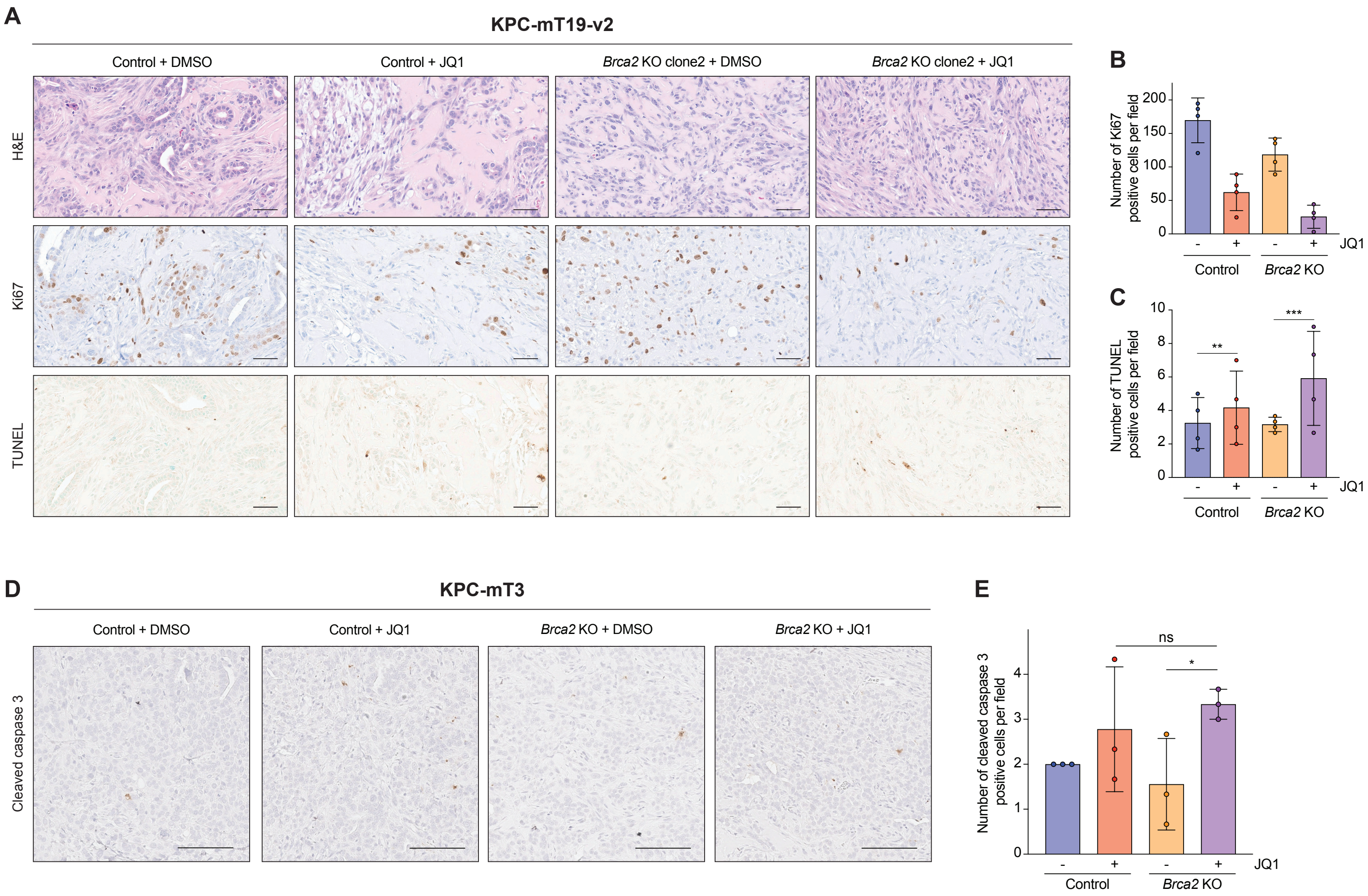

Supplementary Figure 7

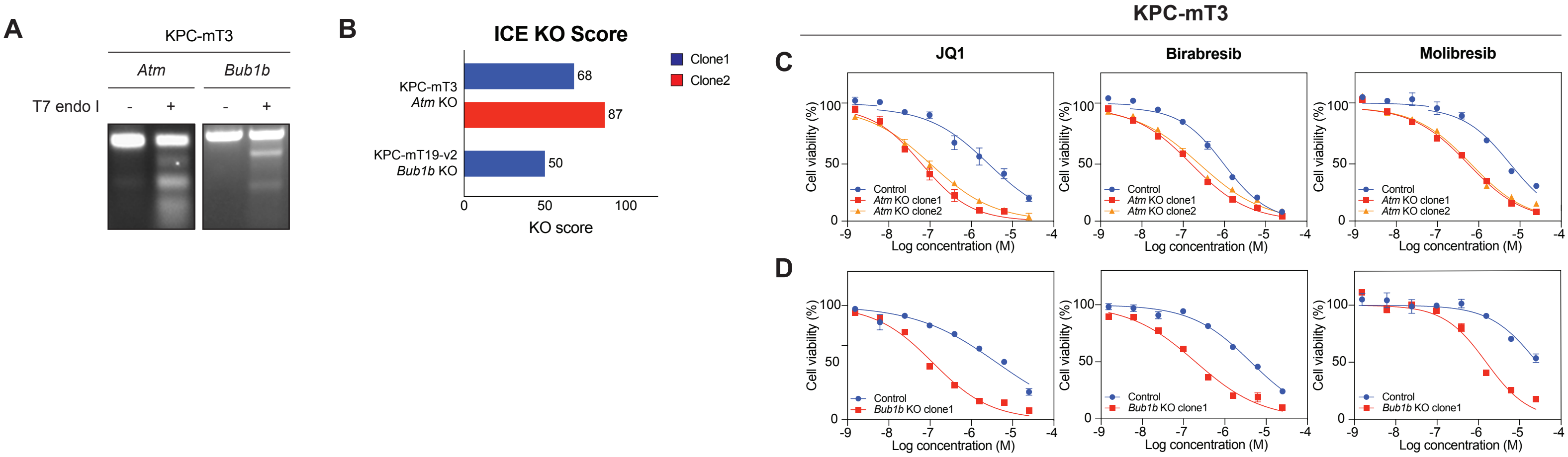

Supplementary Figure 8

A

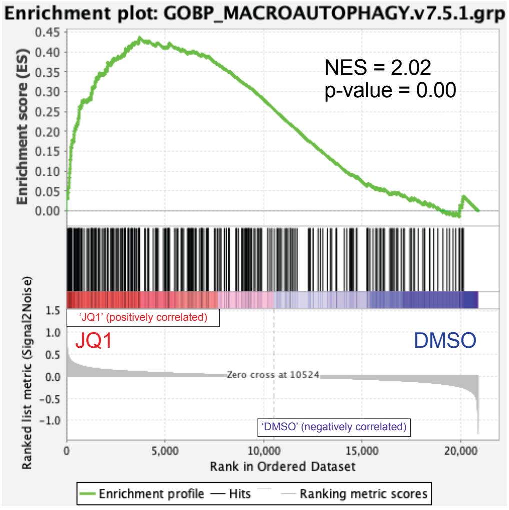

B

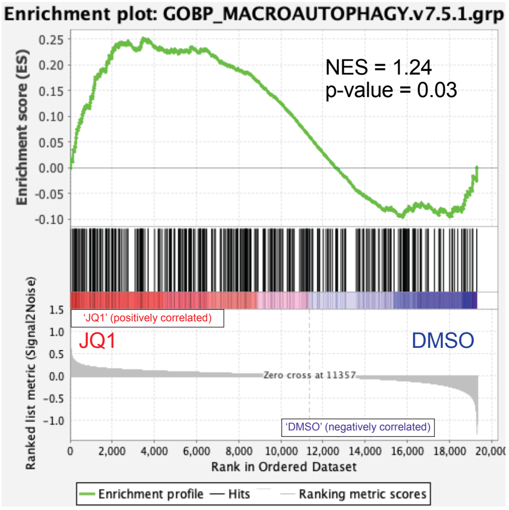

Supplementary Figure 9

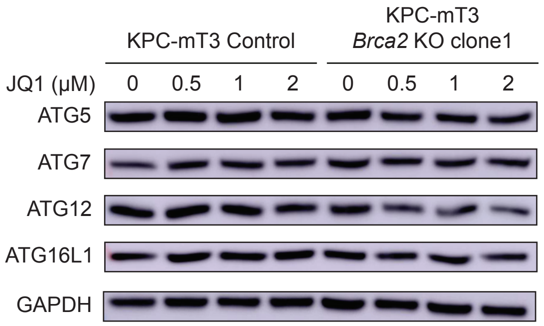

Supplementary Figure 10

A

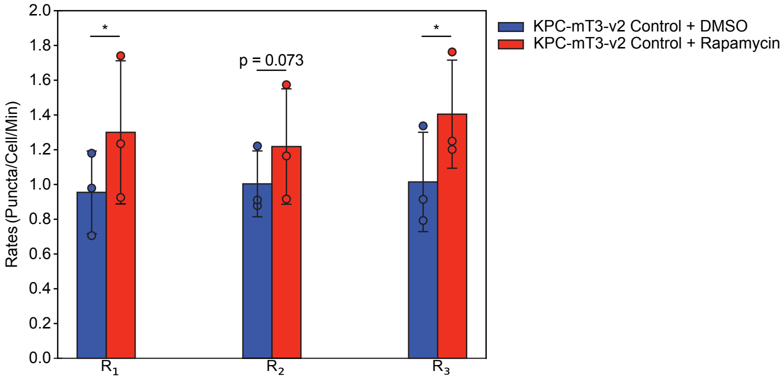

B

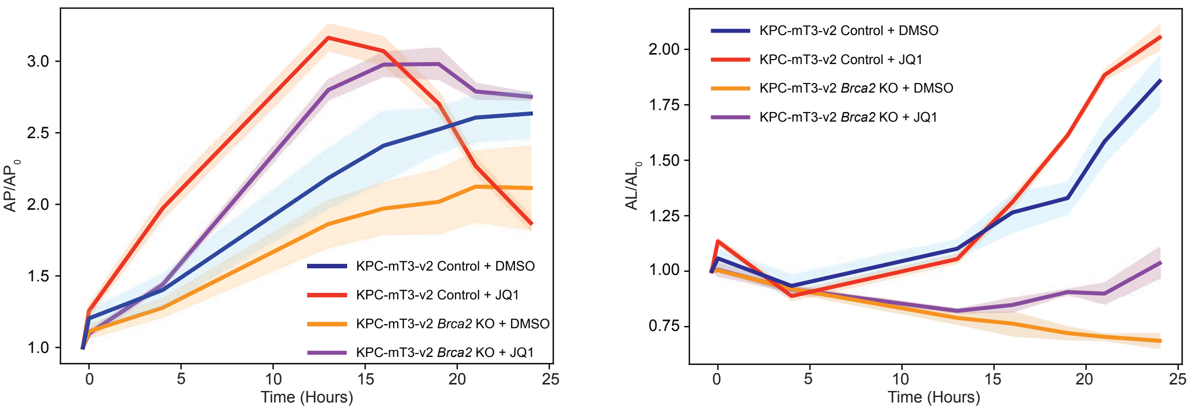

Supplementary Figure 11

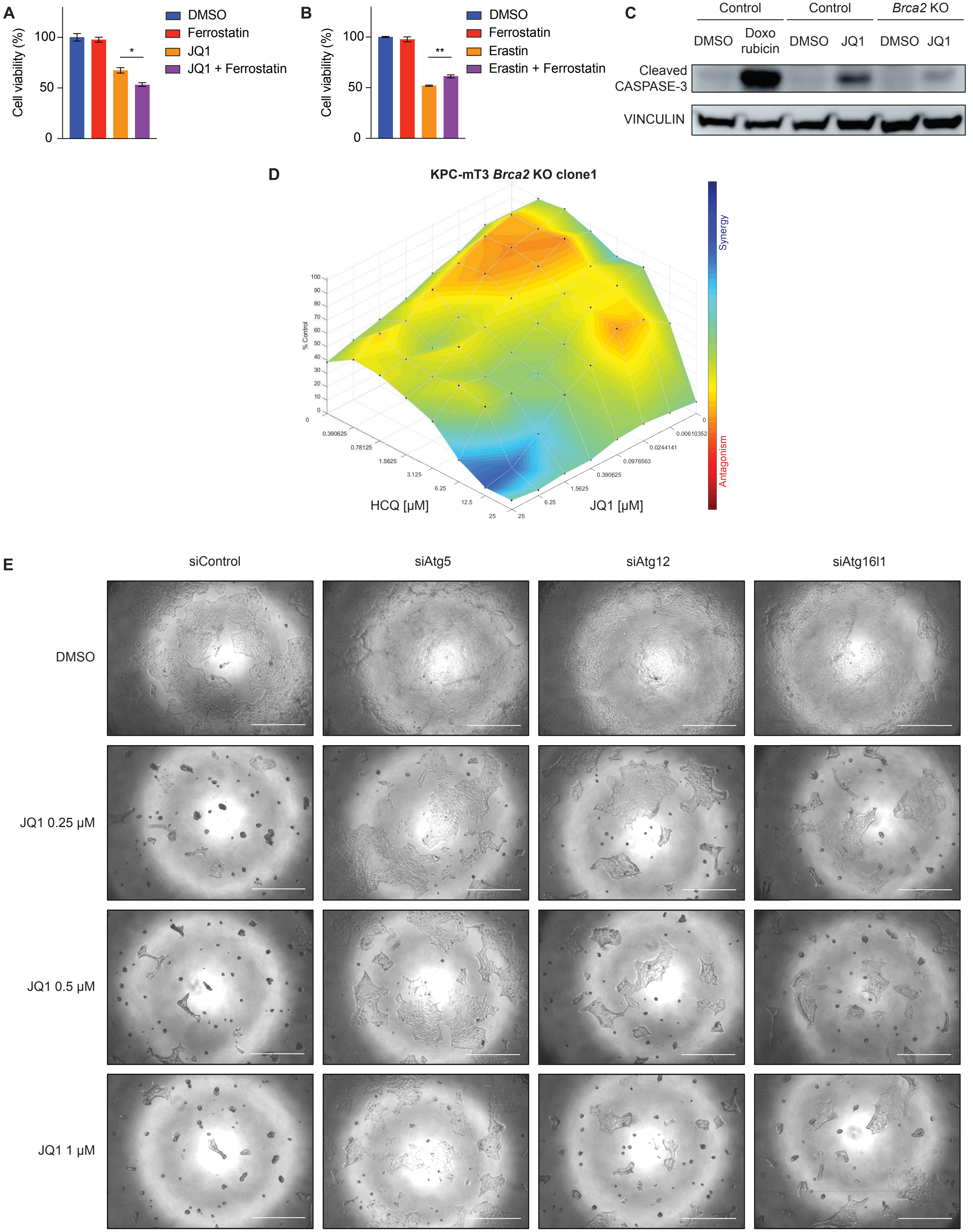

Supplement: Supplementary file 2 — Supplemental Figures [file 41419_2023_6145_MOESM2_ESM.pdf]
