# Supplementary material for: A new vulnerability to BET inhibition due to enhanced autophagy in BRCA2 deficient pancreatic cancer
Source: Cell Death Dis. 2023 Sep 21;14(9):620. doi: 10.1038/s41419-023-06145-9 (PMC10514057; doi:10.1038/s41419-023-06145-9)

Supplemental material to Fig. 5 - Original blots

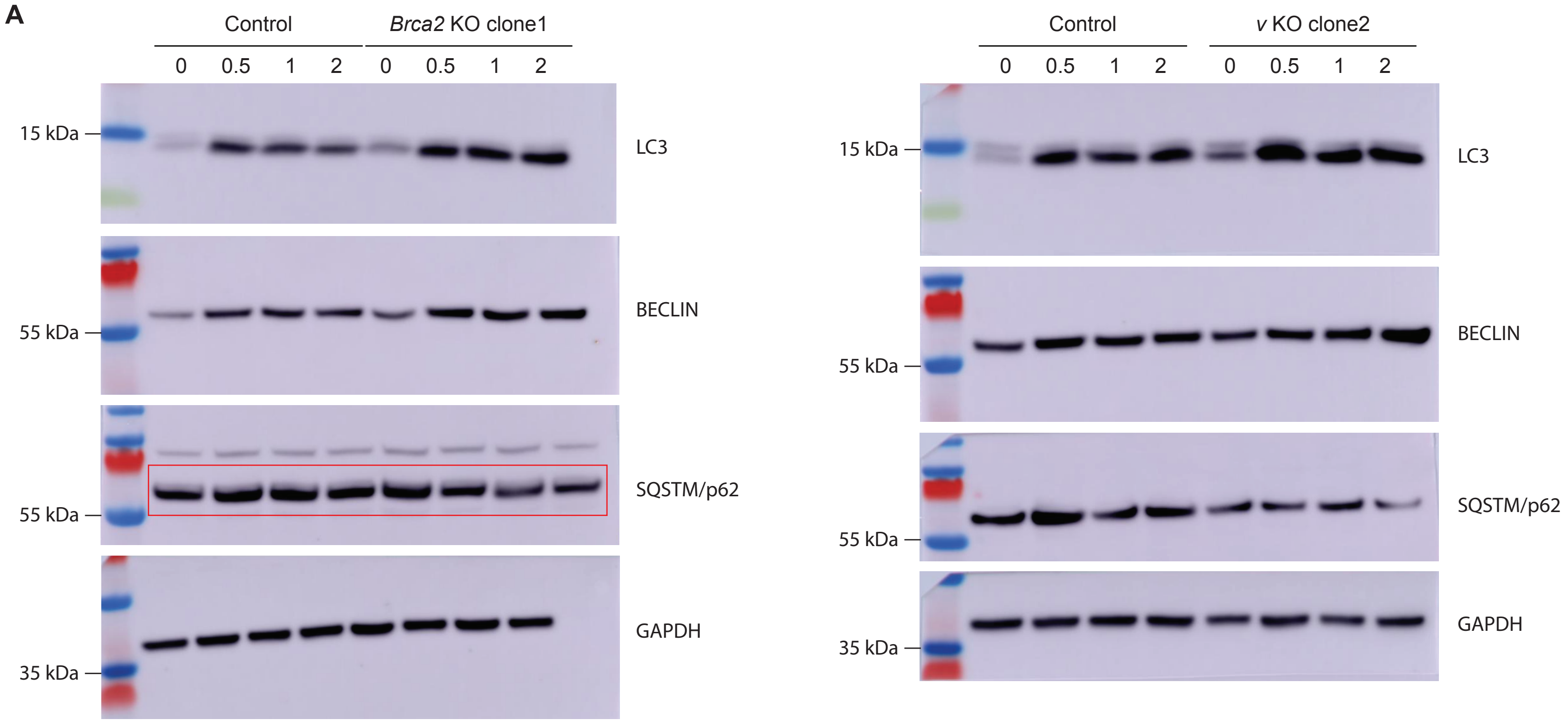

Supplemental material to Fig. 6 - Original blots

E

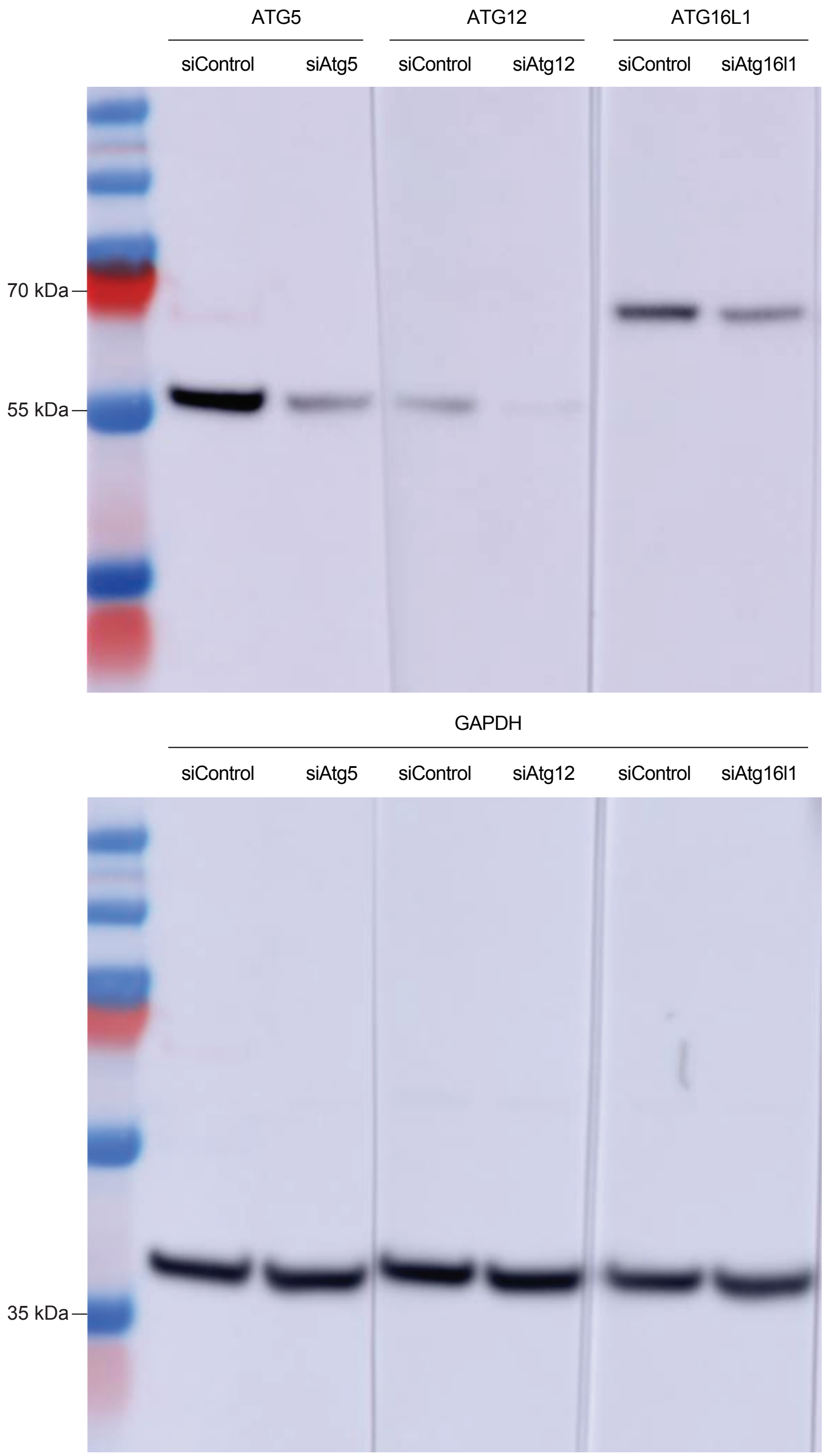

Supplemental material to Supplementary Fig. 4 - Original blots

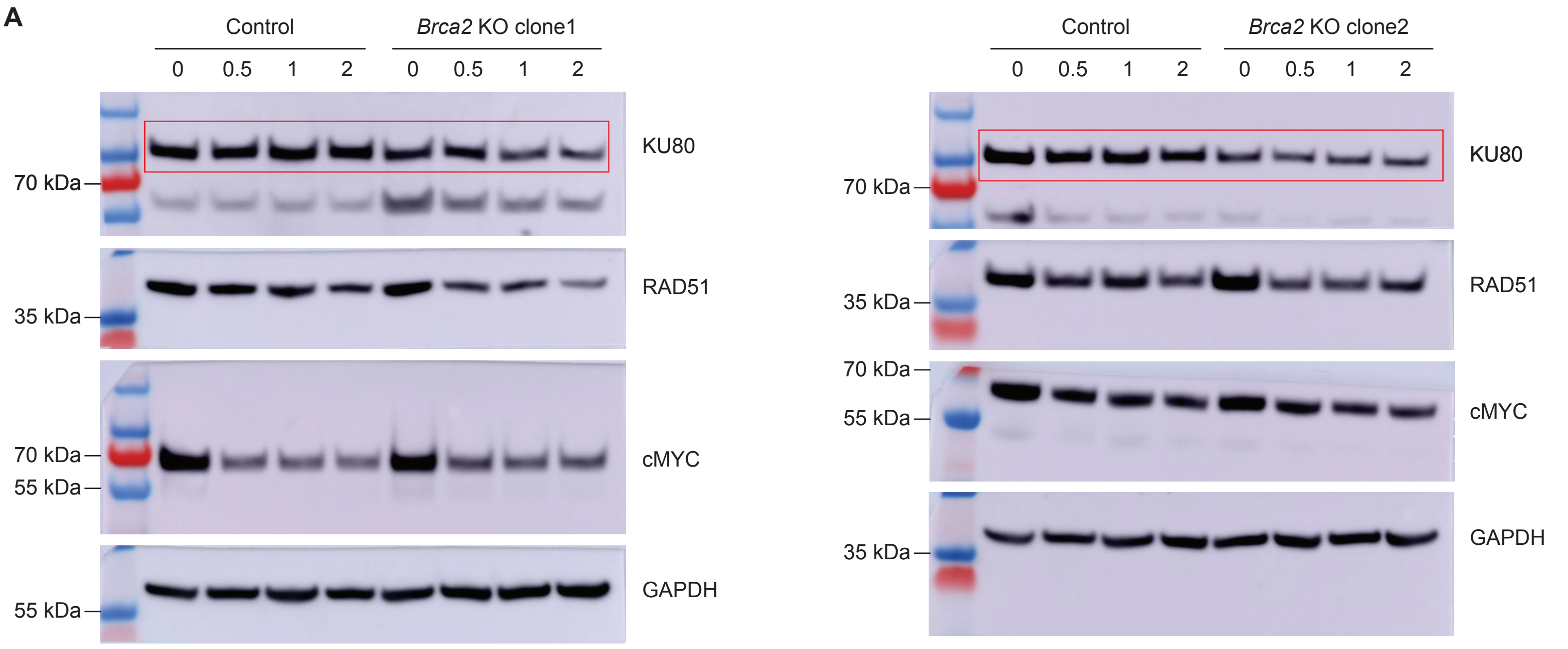

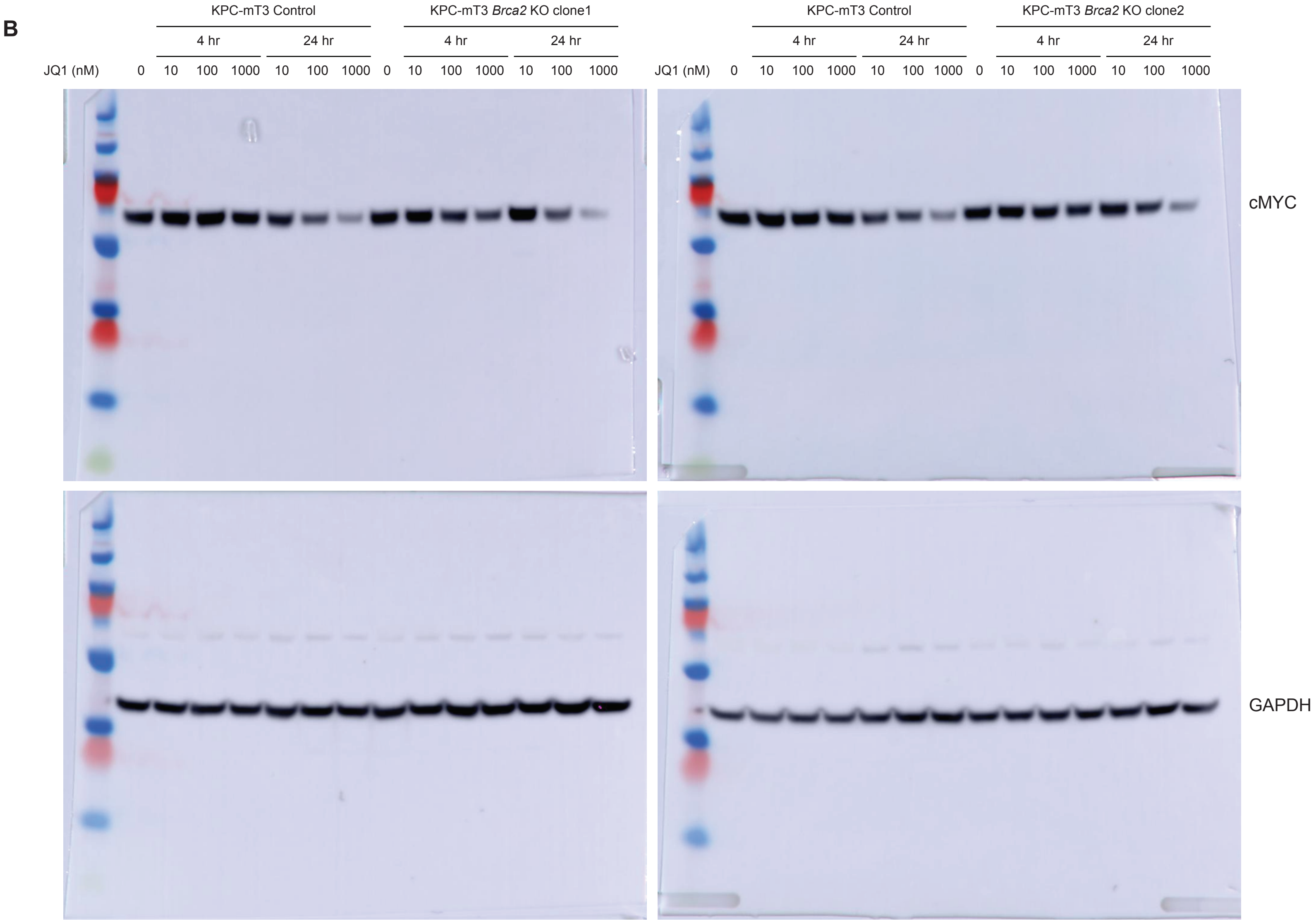

Supplemental material to Supplementary Fig. 9 - Original blots

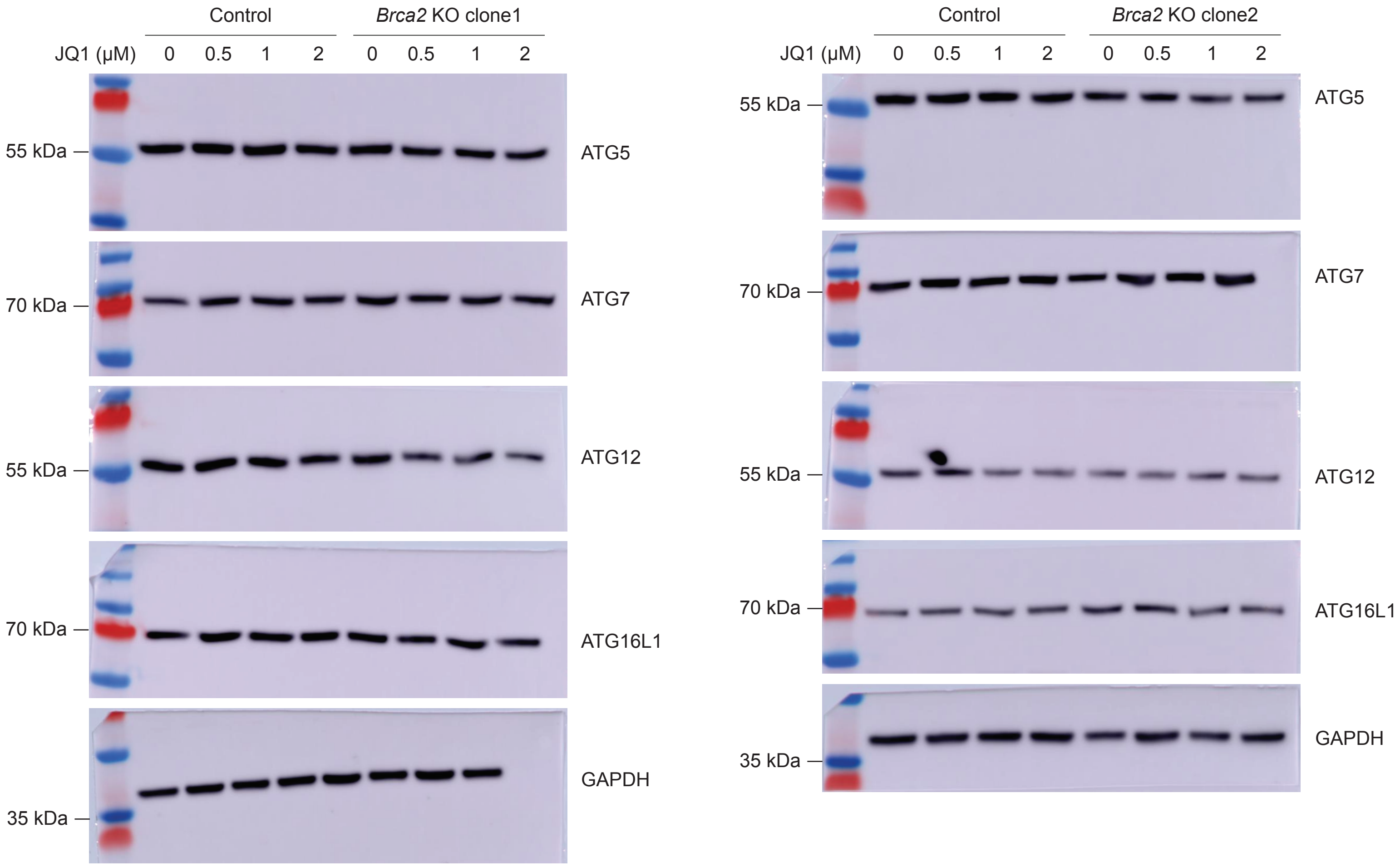

Supplemental material to Supplementary Fig. 11 - Original blots

C

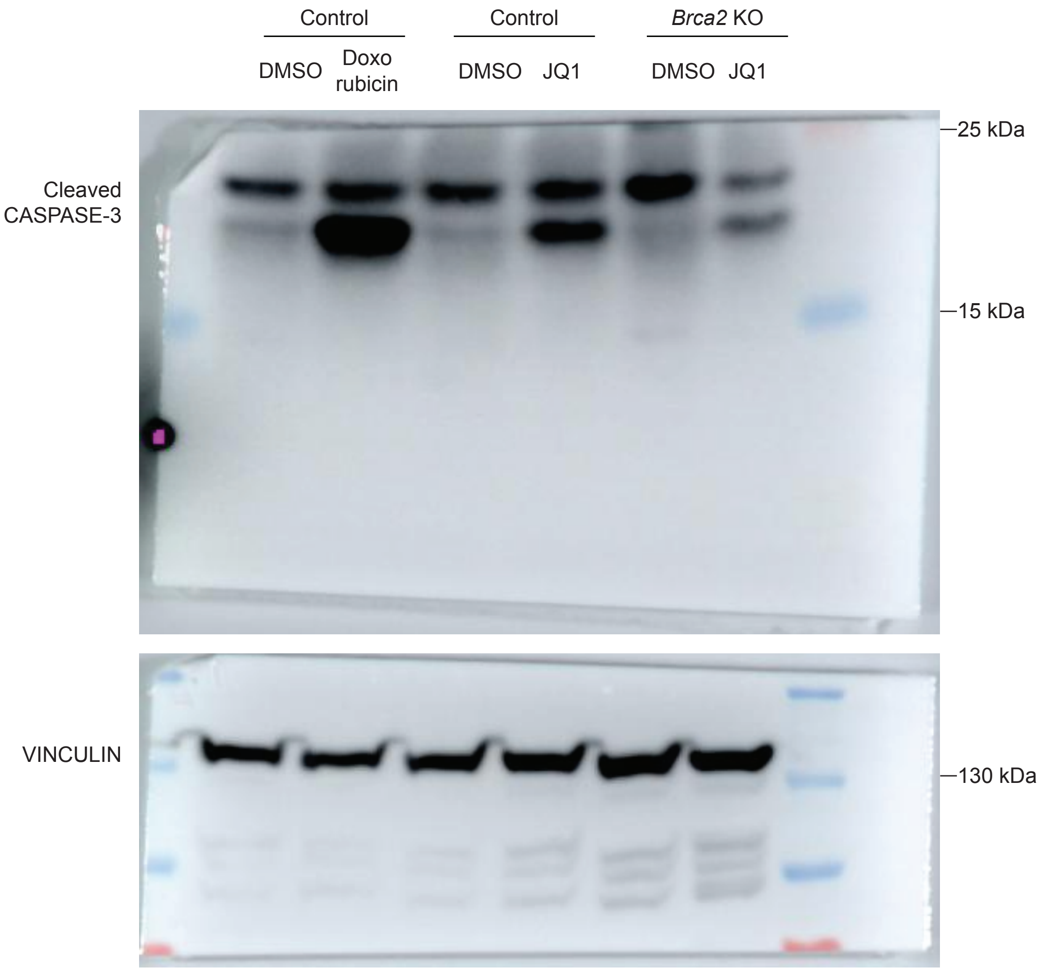

Supplement: Supplementary file 3 — original data files [file 41419_2023_6145_MOESM3_ESM.pdf]
